# Spaced practice and reactive inhibition have limited or no effects on motor sequence learning

**DOI:** 10.1038/s41598-026-52702-5

**Published:** 2026-05-23

**Authors:** Mohan W. Gupta, Timothy C. Rickard

**Affiliations:** 1 Princeton University, Princeton , NJ USA; 2 University of California San Diego, San Diego, CA USA

**Keywords:** Motor sequence learning, Reactive inhibition, Spacing effects, Neuroscience, Psychology, Psychology

## Abstract

Spaced practice in declarative memory tasks consistently yields greater learning than massed practice, but spacing effects are less consistently observed for motor skills. This study evaluates factors that may determine spacing effects on motor skill learning, including: (1) extant theories of declarative spacing effects, (2) reactive inhibition, which transiently impairs performance and may also impair learning, and (3) the micro-consolidation hypothesis, which posits that motor skill learning takes place exclusively during brief performance breaks. Across two experiments, we varied the number of correct sequences per trial and the length of breaks during training, while keeping the total correct sequence count constant, using a widely studied motor sequence task. A pronounced performance advantage was observed for the spaced groups by the end of training. However, on a later test in which the task conditions were equated, group performance was statistically indistinguishable. Hence, spaced practice yielded no or minimal learning advantage and the large reactive inhibition effect in the massed group appears to be a transient performance phenomenon without consequence for learning. Furthermore, we found no evidence for the most straightforward micro-consolidation account, which predicts greater learning with more breaks. Our results are consistent with a simple account advanced by Gupta and Rickard^1,2^, according to which learning occurs entirely online (i.e., concurrently with performance) and is independent of spacing and reactive inhibition. Finally, our findings indicate that proposed mechanisms for declarative spacing effects, such as memory reactivation and contextual variability, do not generalize to motor learning, highlighting fundamental differences between the two learning systems.

## Introduction

There are many reasons one may choose to take breaks while learning a motor skill (e.g., playing the piano); among them are muscle fatigue, cognitive fatigue, and faltering motivation. In the current work, we investigate whether brief breaks may provide not only respite, but also a boost in motor learning.

In the declarative memory literature, there is a positive and potent effect of breaks on learning, a phenomenon known as the *spacing effect*^3–7^. Among the many techniques that have been proposed to enhance declarative learning, spaced practice is one of the more robust. Several of the more promising computational theoretical accounts of the declarative spacing effect may plausibly generalize to motor skill learning, such as accounts based on the ACT-R theory^4^, the Multiscale Context Model^5^, Stimulus Sampling Theory^6^, and the Predictive Performance Eq. 7. Across these models, three core mechanisms of the spacing effect have emerged: encoded memory reactivation, memory contextual diversity, and memory decay. The encoded memory mechanism can in principle be straightforwardly applied to motor skill learning, such that sufficient spacing between bouts of practice causes retrieval and reactivation of a stored motor memory, increasing the strength of the memory, whereas massed practice makes long-term memory retrieval less likely due to the information being held in working memory^8,9^.

Greater contextual diversity has been hypothesized to improve declarative learning by increasing the number and distinctiveness of context–to–item associations that can later cue retrieval^10–13^. A related idea in motor learning is that varying the practice context can promote more flexible, context-independent performance^14–16^. However, the extent to which brief rest breaks create meaningfully different contextual states — and therefore distinct retrieval cues — may be limited in motor tasks due to consistency in task, somatosensory inputs, and effector outputs. This suggests that any spacing benefit from context diversity during short breaks may be limited in a simple motor sequence task.

A third mechanism is memory decay. Declarative memories generally decay relatively rapidly following a power law^17,18^. Due to this decay rate, spacing out practice can supply opportune bumps to the strength of a memory^19^. On the other hand, motor memories tend to decay more slowly over comparable intervals^20–23^. Thus, brief within-session breaks may produce little forgetting and therefore motor learning may benefit less from spaced practice compared to declarative learning.

To our knowledge, the Bayesian Learner model proposed by Körding and colleagues^24^ is the only framework explicitly applied to spacing effects in both declarative and motor skill learning. The model casts learning as multi-timescale adaptation, separating fleeting, fast fluctuations from slower, meaningful changes. In this formulation, a disturbance is any prediction-error–inducing perturbation to the latent skill (i.e., process noise in the updating of the hidden state). Rapid, frequent disturbances — typical of massed practice — are assigned to the fast process and quickly forgotten, whereas more widely spaced disturbances are attributed to the slow process and thus retained. Because timescales are represented as hidden processes in a linear dynamical system, the learner uses temporal structure to decide whether a given disturbance reflects noise (fast) or a stable change (slow). Applied to our experiments, the longer inter-practice intervals in the spaced groups should shift credit to the slow process, enhancing retention and performance.

**Fig. 1:**
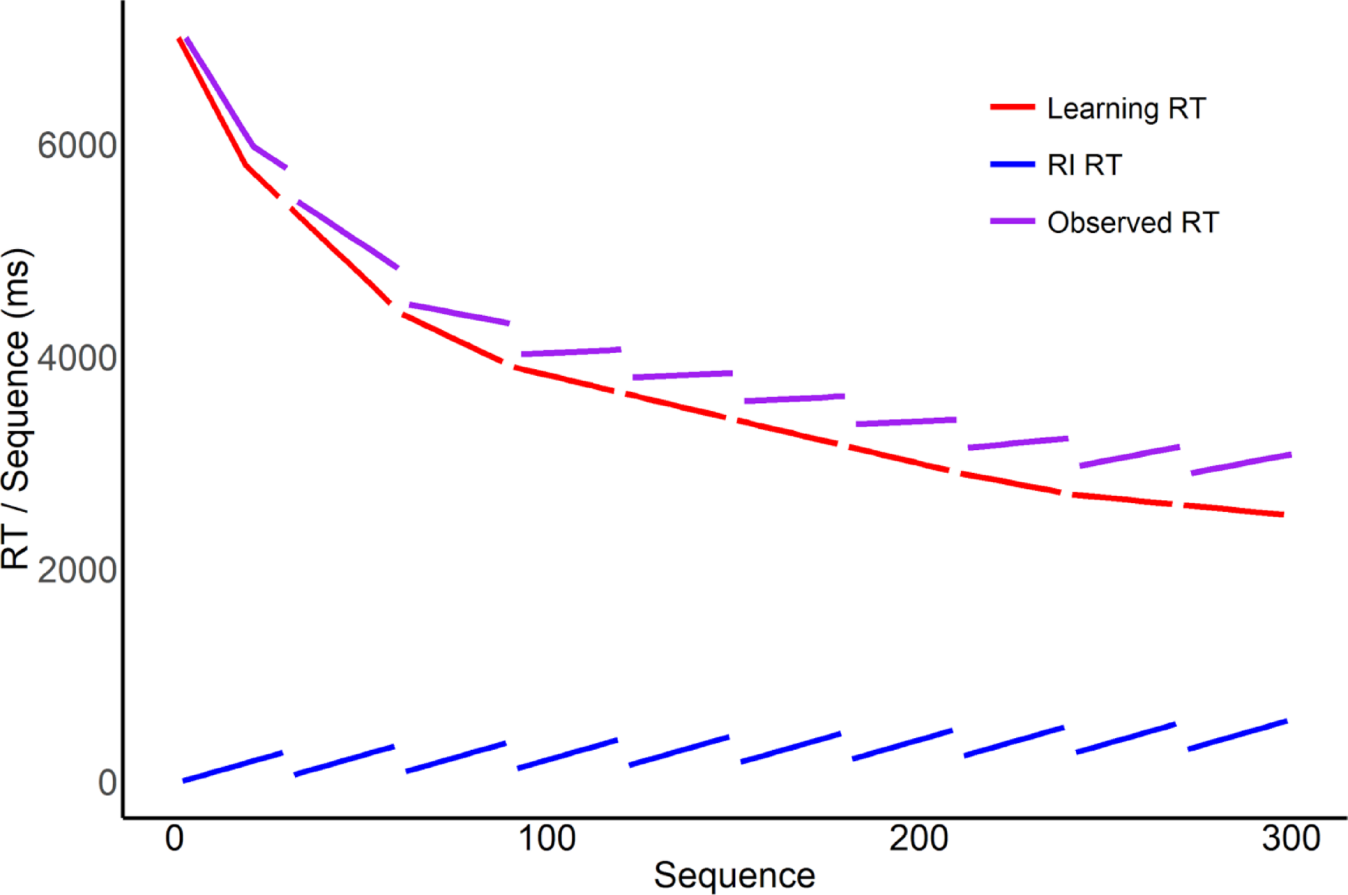
Conceptual simulation illustrating how observed performance can reflect the sum of learning-related improvement and RI. The red curve (“Learning RT”) shows how sequence RT declines with practice in the absence of RI. The blue curve (“RI RT”) shows the accumulation of RI within and across trials, and partially dissipates during breaks. The purple curve is the resulting “Observed RT” (red + blue). Early in training, learning outweighs RI, producing within-trial performance gains; later, RI can dominate and produce within-trial worsening despite ongoing learning. The dissipation of RI during breaks gives the illusion of offline learning. This schematic illustrates performance across successive sequences and is not intended to depict the training/test phases of the experiment.

Beyond theories of the declarative spacing effect, two hypotheses about motor learning suggest that spacing may enhance learning. Reactive inhibition (RI) is a well-established empirical phenomenon of worsening motor performance during continuous practice which dissipates during breaks^1,2,25–28^ (Fig. 1; for a candidate neurological mechanistic account of RI, see Bächinger and colleagues^29^. RI is more pronounced when practice is sustained for a long period (massed practice), but less pronounced when practice is short with frequent breaks (spaced practice). Although the classic effect of RI is a transient worsening of performance, several studies have addressed the possibility that RI may also negatively affect motor learning. Across alphabet printing, peg board learning, the stabilometer task, and the Tsai-Partington numbers tasks, every possible result has been found; greater learning with spaced training^30,31^, no learning difference between massed and spaced training^32,33^, and greater learning with massed training^34^. In a meta-analysis, Lee and Genovese^35^ concluded that massed practice impairs both performance and learning. However, given the variety of findings, methodological limitations, and small sample sizes in some cases, we view that literature as inconclusive. If RI does attenuate motor learning, then reducing it through spacing would yield enhanced learning and performance on a delayed test.

The new theory of *micro-consolidation* posits that motor learning occurs primarily or exclusively during breaks (offline), driven by hippocampal to neocortical replay of learned sequences on the time scale of seconds^36–39^. This theory is akin to the proposed encoded memory reactivation mechanism of the spacing effect in declarative learning. Hence, frequent breaks between brief performance trials (spaced practice) should enhance learning due to greater memory reactivations. In apparent support of this theory, motor sequence performance immediately after a break is often better than at the end of the preceding training trial^36,37,39^.

Finally, there is the possibility that spaced practice has no effect at all on motor learning, as proposed in our previous work, Gupta and Rickard^1,2^. We proposed an online-learning account where learning occurs during sequence execution, whereas breaks only modulate performance by allowing RI to dissipate. As illustrated conceptually in Fig. 1, the red curve corresponds to the underlying skill improvement that would be expressed in the absence of RI, whereas the observed performance (purple) reflects this learning component plus an added RI-related slowing term (blue). From this perspective, massed practice will have worse performance by the end of training because RI accumulates, but once RI is removed (e.g., after rest and under matched task conditions), performance would be equivalent across groups when the amount of practice is matched. This differs from micro-consolidation and domain-general spacing accounts, which predict that spaced schedules should yield superior retained performance because learning is strengthened during breaks and/or via reactivation and contextual variability.

The question of whether spacing effects occur in the current motor sequence task is also informed by several empirical studies of other motor tasks. Spacing effects have been observed for several tasks, including the rotary pursuit test, typing, discrete linear movement learning, computer game learning, dynamic balancing, and motor sequence learning^40–46^. In contrast, investigations into other complex motor skills such as cup stacking and piano learning^47,48^ and simple sequence learning in both humans and monkeys^49^ have shown either a lack of spacing effects or inconsistent benefits across measured variables. Thus, prior work does not provide clear guidance on what to expect in the current experiments. Further, there appear to have been no prior studies focused on investigating spacing effects for the finger tapping task investigated here, which has dominated the extensive literature on the hypothesis of sleep-based motor sequence memory consolidation over the last three decades^50^.

We investigated whether spacing and RI affect motor sequence learning by manipulating both trial length (the number of sequences within each trial) and the duration of breaks between trials. In Experiment 1, both groups learned to perform a five-item (five keypress) sequence. The *spaced* group performed five correct sequences during each of the 35 trials with 30 s breaks between trials. The *massed* group performed 25 correct sequences during each of the seven trials with 10 s breaks. After a 15-minute rest period during which the cumulative effect of RI over training trials is expected to fully resolve, there was a test involving an identical spaced task for both groups, with five correct sequences per trial and 30 s breaks. The total number of training and test sequences was the same for the two groups. Because both groups performed the same task on the test, any learning and RI effects across test trials should be equated. Hence, any performance difference between groups on the test should exclusively reflect differences in the amount of learning during training. In Experiment 2, we increased the sequence length to nine-items. The spaced group performed four correct sequences per trial with 30 s breaks, while the massed group performed 12 correct sequences with 10 s breaks. All other design aspects mirrored those of Experiment 1.

Based on prior results^1,2^, the effect of RI on performance in the spaced group should be limited primarily to sequences within individual trials, with minimal RI accrual across trials (i.e., each 30 s break should be sufficient to dissipate most of the RI that accrued during the preceding trial). However, for the massed group there should be substantial accrual of unresolved RI across trials. Hence, if RI impairs learning in a dose-response manner, we should see better final test performance in the spaced group (a spacing effect). In addition, because there is a much longer total break time across training in the spaced group (1,020 s, vs. 70 s in the massed group), there is more opportunity for offline micro-consolidation in the spaced group, again suggesting a positive spacing effect.

In summary, we should observe better test phase performance in the spaced group if RI impairs learning and (or) if spaced training enhances learning through other spacing mechanisms, including micro-consolidation. In contrast, we should observe equivalent test performance in the two groups if each of the following conditions are true: (1) RI is exclusively a performance phenomenon, (2) the greater break frequency and duration for the spaced group in does not promote more offline micro-consolidation, and (3) none of the spacing mechanisms in theories developed for declarative learning generalize to the case of single-session motor sequence learning. The latter possibility is consistent with the hypothesis advanced by Gupta and Rickard^1,2^.

## Results

### Errors

We quantified errors for each participant as the number of incorrect keypresses made before each correct sequence within a trial. During training, the groups were similar in Experiment 1 (spaced: 0.38 incorrect keypresses per correct sequence, massed: 0.41), with evidence favoring no difference (BF_01_ = 5.25, d = − 0.056). In Experiment 2, however, the massed group made more training errors than the spaced group (massed: 1.73, spaced: 1.26), with evidence for a difference (BF_10_ = 5.08, d = 0.282).

In the test phase, where both groups performed the spaced version of the task, the spaced group made more errors in both experiments than the massed group (Experiment 1: 0.44 vs. 0.17, BF_10_ = 8.28, d = − 0.32; Experiment 2: 0.99 vs. 0.60, BF_10_ = 8.0, d = − 0.30). This pattern raises the possibility of a test-phase speed–accuracy trade-off in the spaced condition: relative to the massed group, the spaced group may have prioritized faster responding during the test, with a corresponding increase in errors. At the same time, the error results do not support a single, consistent speed–accuracy trade-off explanation across training and testing. During training, errors were indistinguishable in Experiment 1 and were lower for the spaced group in Experiment 2.

### Within-trial RI

To test whether RI accrued over the course of a trial, we fit a Bayesian linear mixed-effects model predicting time to execute an entire correct sequence in milliseconds (RT) from within-trial sequence number, group, and their interaction, with random intercepts and slopes by participant and a random intercept for trials nested within participant. RT reliably increased across sequences within a trial in both groups. In Experiment 1, RT increased with sequence number in the massed group (BF_10_ = 3.09e^57^, β = 5.09 ms/sequence) and also in the spaced group (BF_10_ = 4.64e^8^, β = 23.33 ms/sequence). The same pattern held in Experiment 2: RT increased robustly with sequence number in the massed group (BF_10_ = 2.85e^13^, β = 58.24 ms/sequence) and in the spaced group (BF_10_ = 2.09e^8^, β = 62.18 ms/sequence), consistent with within-trial performance slowing as participants progressed through successive correct sequences.

### Training RTs

**Fig. 2:**
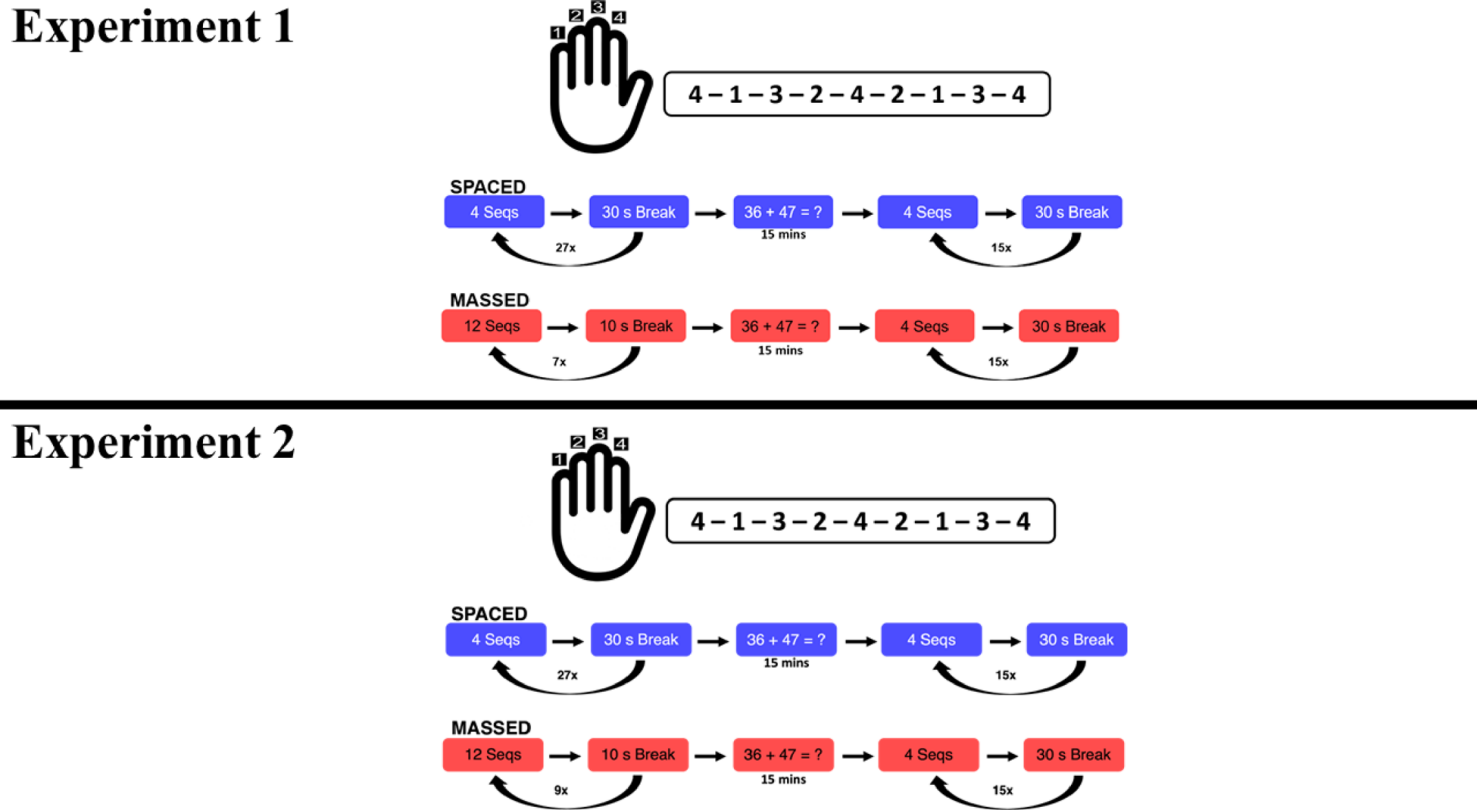
In both experiments, participants learned to type a motor sequence over one session with their non-dominant left hand. The spaced groups performed fewer sequences per trial and had longer breaks than the massed groups. The overall number of sequences performed was equated between groups, but break time was significantly longer in the spaced group. After training, participants performed 15 min of double-digit addition during the rest period. After, both groups were tested on the trained sequence with the spaced design for sequences per trial and break time.

Next, we asked whether spacing affected end-of-training performance. In both experiments, the massed group finished training with longer RTs than did the spaced group. In Experiment 1, comparing mean RTs over the last 25 training sequences, we found strong evidence for a group difference (BF_10_ = 6243.1, d = 0.54), consistent with greater cumulative RI in the massed condition (Fig. 2a). Experiment 2 showed the same pattern, again with overwhelming evidence for a group difference on the last 25 training sequences (BF_10_ = 12.66e^76^, d = 0.68; Fig. 2c).

### Post-rest gain

To test whether the change from the end of training to the test phase differed by group, we computed a within-participant difference score: mean RT at the end of training minus mean RT at the start of test (Experiment 1: last 25 training sequences minus first 25 test sequences; Experiment 2: last 24 minus first 24). In both experiments, this training-to-test shift was larger in the massed group than in the spaced group, consistent with the idea that massed performance at the end of training is disproportionately slowed by RI and performance rebounds after the rest period. In Experiment 1, the group difference in these difference scores was strongly supported (BF_10_ = 2e^5^, d = 0.68; Fig. 2a). Experiment 2 showed the same pattern with similarly strong evidence (BF_10_ = 7.95e^14^, d = 1.0; Fig. 2c).

### Final test RTs

The key test of spacing effects on learning involved comparison of the mean RTs on correct sequences during the test phase. This comparison was designed to isolate learning rather than transient performance factors: participants completed a rest period intended to allow accumulated RI from training to dissipate, and the test phase used the same spaced structure for both groups. Thus, any RI that accrues during testing, and any additional learning that occurs during testing, should be equivalent across groups. Under this equated test context, we found no statistical evidence for an RT difference between groups. In Experiment 1, the Bayes factor weakly favored the null (BF_01_ = 2.76, d = 0.14; Fig. 2b), and Experiment 2 showed the same pattern (BF_01_ = 2.51, d = 0.15; Fig. 2d). Importantly to note, the RT difference that was very apparent at the end of training all but disappeared.

## Discussion

We investigated whether spaced practice enhances motor skill learning in two experiments. During training in both experiments, a larger amount of RI was induced in the massed group by limiting the number and duration of breaks, and by requiring participants to complete a relatively large number of correct sequences per trial. After a 15-minute rest to dissipate RI, both groups performed the spaced task from the training phase. Despite the large performance differences at the end of training in both experiments, test performance was statistically equivalent in the two groups, suggesting that neither RI, micro-consolidation, nor other candidate mechanisms for spacing effects influenced training phase learning.

In both experiments there was a non-significant effect (*d* = 0.14; d = 0.15) toward better final test performance in the spaced group, and thus we cannot rule-out a small advantage in learning for that group. It is also possible that the 15-minute rest period was insufficient to completely dissipate RI. However, that possibility seems unlikely given that on average the training session lasted around seven minutes, not including breaks, with the 15-minute rest period more than doubling that duration. Further, participants had the same number of total correct keypresses within each experiment. Regardless, any difference in achieved learning between the two groups is at best minimal. That apparent absence of a spacing effect was surprising in light of previous evidence that learning during spaced training is superior to learning during massed training, not only in declarative memory tasks, but also in some types of cognitive skill learning^51,52^.

The conclusions above are based solely on correct sequence RT. Error rate differences between groups should also be considered. In particular, the spaced-trained group tended to make more errors on the equated test, even though RTs were numerically (slightly) faster. As noted earlier, this pattern leaves open a test-phase speed–accuracy trade-off: if some participants prioritized speed under the spaced test demands, their faster correct-sequence RTs could coexist with elevated errors, producing a small RT advantage without implying stronger learning. Nevertheless, error-rate patterns during training were not consistent across experiments, which makes a single, global speed–accuracy explanation unlikely. Given these considerations, we interpret the most robust aspect of the data as follows: the large end-of-training RT gap—driven by RI under massed training conditions—largely disappears after the rest period and under an equated test schedule. This pattern is consistent with the online learning account advanced by Gupta and Rickard^1,2^ in which learning occurs through the execution of sequences rather than during breaks, and the spacing manipulation mainly affects performance via RI accrual and dissipation. On this view, any remaining group difference at test is small and could plausibly reflect residual RI or a modest speed–accuracy trade-off rather than a spacing-based enhancement of learning.

Our findings place boundary conditions on the micro-consolidation account of learning. First, if all of the training phase learning occurred during breaks, then the amount of learning that occurred during each break must have been substantially greater in the massed groups (e.g., with eight 10 s breaks) than in the spaced groups (e.g., with twenty-seven 30 s breaks), given the equivalent performance on the test. The micro-consolidation theory, as developed to date, does not specify whether or how the rate of offline consolidation depends on either the number of sequences practiced within each trial or the time available during each break, and it remains to be seen whether it can accommodate the current results. Of relevance here, Buch and colleagues^39^ observed that hippocampal activity associated with offline micro-consolidation occurred throughout the entire 10 s break periods in their study. Hence, if there is a dose-response relationship between break time and amount of micro-consolidation, the spaced groups should have shown greater overall learning. Instead, our results align more closely with recent evidence from Das and colleagues^53^, who showed that short rest periods in a similar finger-tapping task produce micro-offline performance gains during training but no lasting advantage in later tests relative to continuous practice. They further demonstrated that such gains are present even for random, nonrepeating sequences, where no micro-consolidation could occur.

Our results further suggest that the mechanisms proposed in extant spacing effect theories in declarative learning do not extend to motor skill learning, at least for the current finger tapping task. In particular, these results are inconsistent with the Bayesian Learner model^24^, which predicts domain-general spacing benefits. Other theories of the declarative spacing effect posit that spacing benefits learning through the reactivation of memories, strengthening the original memory itself, or associations between items and contexts, like in the Multiscale Context Model^5^ and the Predictive Performance Equation models^7^. This reactivation may be particularly effective when there is sufficient spacing between repetitions, possibly due to memory traces being primarily mediated by the cortex and out of working memory^11,23^. However, reactivation may not benefit motor skill learning in the same way as declarative memories, possibly due to differing neural computations in areas like the basal ganglia and primary motor cortex^54^. Further, motor memories are much more resistant to forgetting^20,55^ and thus reactivation may only give a marginal benefit to the memory trace strength, resulting in minimal spacing effects.

Along with memory reactivations, spaced practice in declarative learning is thought to increase contextual variability, where each spaced repetition occurs in a distinct temporal or environmental context, enhancing memory strength through contextual reinstatement and context diversity^5,12,56^. In contrast, motor skill learning may be more influenced by physical and sensorimotor context variability, such as changes in movement patterns, speed, or force application^57,58^. While spacing might alter the temporal context, we speculate that it does not influence the internal sensorimotor context that drives motor memory formation and retention. As a result, the limited ability of spacing to modify these critical features may constrain its impact on motor learning. Instead, other forms of variability (e.g., interleaved practice schedules) have yielded more robust effects on motor skill retention and transfer^59–62^.

Our findings mirror earlier studies comparing massed and spaced practice with sleep during the retention period^26,27,50,63^. Once confounding factors such as circadian phase and data averaging are controlled for in those studies, there is little evidence that either massed or spaced training produces overnight improvements (or decrements) in performance. In our study, we likewise observed equivalent post-rest performance across groups. Importantly, we do not take these results as evidence against consolidation as memory stabilization (i.e., preserving skill over time). Rather, the convergence across studies suggests that, for simple motor sequence tasks at least, spacing and sleep may confer limited memory-strength enhancement—that is, they do not reliably yield additional performance gains beyond what is achieved through practice itself.

Our results and others^49,53^ suggest that spaced practice confers little or no benefit in relatively simple motor sequence learning tasks. Nevertheless, there is ample evidence that declarative processes contribute to motor learning^64–67^. Consistent with this, motor sequence learning can recruit hippocampal/medial temporal lobe processes, especially early in learning^36–39^. The benefits of spaced practice on motor learning may depend on the stage of learning it targets (e.g., early declarative-supported learning vs. later procedural refinement), and this timing is likely task specific. For example, spacing may confer greater benefit to more complex motor skills that have a protracted declarative learning phase, such as driving or typing. This possibility points to an important direction for future research on spacing: identifying the time course of declarative involvement in motor learning and determining whether and for which types of tasks spacing influences that component of learning.

Possibly, the short time frame of the current experiments (~ 30 min) was insufficient for spacing to produce a noticeable benefit in performance. For declarative memory, the largest effects are often found when spacing between sessions spans days and the final test is also days after the last training session^5,19^. However, in a meta-analysis of that literature by Cepeda and colleagues^11^, modest spacing effects were found for declarative tasks when spacing was similar to that in the current tasks (between 1 and 59 s between trials with retention intervals between 1 and 10 min), suggesting that our conclusions are not idiosyncratic to our timeframe. The possibility that spacing effects for motor tasks are more robust at longer time intervals does not undermine our conclusion that RI is independent of motor skill learning, nor does it contradict our conclusion that these results constitute a substantial challenge to the micro-consolidation account.

One limitation is that our design does not include a random-sequence control, so we cannot directly dissociate sequence-specific learning from nonspecific improvements in motor execution fluency. Participants can improve on both repeating and random sequences in sequence-learning paradigms, although gains are typically much larger for repeating sequences, making a pure fluency account of sequence-learning curves unlikely. However, nonspecific fluency could still contribute to performance and, in principle, could obscure small group differences. Our primary inference is therefore anchored in the equated post-rest test, where both groups perform the same spaced task structure; any general fluency gains accrued across the session should contribute to performance in both groups at test, reducing (though not eliminating) the likelihood that our null reflects only nonspecific fluency. Future work that includes random-sequence probe blocks (or an explicit random-sequence control group) would allow these components to be separated directly and could reveal whether any small advantage is specific to sequence learning.

Our results raise the possibility that massed motor skill training, and the associated RI, is not detrimental to naturalistic motor sequence learning – such as training on a musical instrument or sport – even when the worsening of performance due to RI is palpable. However, there may be factors that limit the generalizability of that conclusion. Our experiments probe learning over short timescales, and procedural skill acquisition often unfolds over much longer periods; spacing benefits might therefore be more likely to emerge with multi-session training and/or longer retention intervals (e.g., days). Nevertheless, in accordance with our results, Wiseheart and colleagues^47^ reported that spaced practice in piano learning was not reliably more beneficial than massed practice.

We do not claim that massed practice could be extended indefinitely without introducing factors that may affect learning (e.g., attentional lapses, motivational decline, physical fatigue, or escalating errors). In the current tasks, error rates were not systematically higher in the massed groups, but longer trial durations coupled with higher error rates may shift the learning balance, suggesting an learning optimization trade-off consistent with recent evidence for an optimal error rate in motor skill learning^68^.

In conclusion, our findings suggest that models of the spacing effects for declarative tasks do not generalize to motor sequence learning. Instead, our results align with the online learning account, wherein motor learning occurs during performance and is unaffected by breaks or RI. This result places important boundary conditions on existing theories, such as the Bayesian Learner model and micro-consolidation accounts, and highlights the need to better understand the unique mechanisms that underlie effective motor skill learning.

## Methods

### Participants

To determine that sample size for Experiment 1, we conducted an a priori power analysis using G*Power^69^. Based on the final-test difference between spaced and massed practice reported by Gupta and Rickard^2^— with α = 0.05, power = 0.80, effect size d = 0.49, and a two-tailed independent-samples t-test, the required sample was 163 participants. We recruited 171 right-handed participants: 85 in the massed group (M age = 20.8 years, 76.5% female) and 86 in the spaced group (M age = 20.1 years, 81.4% female). Following the data-cleaning procedure described in Data Analysis and Cleaning, eight participants were excluded (six massed, two spaced), yielding a final sample of 163.

In Experiment 2, we aimed to recruit a greater number of participants in order to replicate Experiment 1. We recruited 196 right-handed participants, 98 in the massed group (age = 20.5, F = 73.4%) and 98 in the spaced group (age = 21.1, F = 76.5%). Ten participants (six from the spaced group and four from the massed group) were removed using the cleaning procedure described in Data Analysis and Cleaning, leaving 186 participants.

The experiment was conducted online and participants were recruited from the University of California, San Diego SONA undergraduate participant pool. Participants provided informed consent via button press prior to participation. All procedures were approved by the Institutional Review Board of the University of California, San Diego, and were conducted in accordance with relevant guidelines and regulations and in compliance with the Declaration of Helsinki.

### Experimental design and procedure

**Fig. 3:**
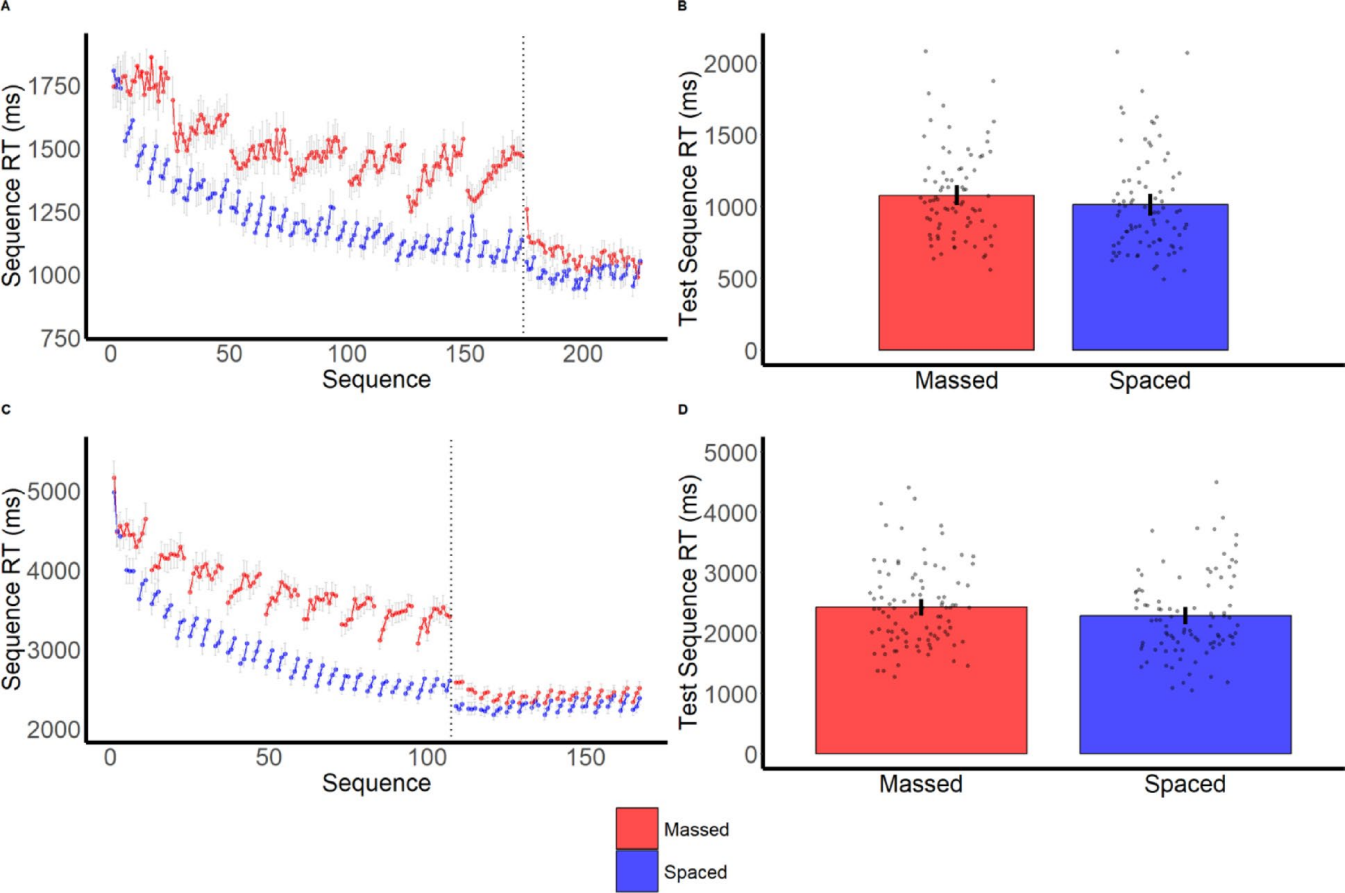
(**a**) Experiment 1: Each dot is the mean RT over subjects for a correctly completed sequence; line segments connect sequences within the same trial. Because the spaced group completed more trials, more first-sequence warmup observations were removed, so fewer sequences are shown. Gray error bars indicate SE. (**b**) Experiment 1: Final test mean RT averaged across test trials. Dots show participant means; error bars are 95% CIs. (**c**) Experiment 2: Same plotting conventions as in (a). (**d**) Experiment 2: Same plotting conventions as in (b).

Participants performed a classic finger-tapping-task in which they repeated the sequence, 4-1-3-2-4, as quickly and accurately as possible with their non-dominant left hand^70^ (Fig. 3). Throughout the experiment, the numbered key sequence was displayed horizontally on the computer screen. Each keypress response was indicated by adding a ‘*’ to a single row underneath the displayed sequence. This did not indicate if the response was correct or incorrect, only that a key was pressed. Between trials, a displayed countdown timer indicated how much break time was left. Participants performed one correct warmup sequence before starting the main task. They had 10 s to do so or had to restart the warmup trial. The warmup trial was not included in the analyses.

A between-participant design was used, in which the massed group completed 25 correct sequences per training trial with 10 s breaks between trials and the spaced group completed 5 correct sequences per training trial with 30 s breaks. After 175 completed sequences during the training phase in both groups, there was a 15-minute rest wherein participants in both groups performed a distraction task of double-digit addition. During the subsequent test both groups performed 10 trials with 5 correct sequences per trial and 30 s breaks.

In Experiment 2, participants performed the same task as in Experiment 1, however, the sequence length was increased to nine-items, 4-1-3-2-4-2-3-1-4 (we reversed the first four items of the original sequence and then appended it to the end). We used a longer sequence to reduce sequence RT variance (by averaging over more items in a sequence reduces variability) and to slow the learning rate relative to the 5-keypress sequence. Participants in the massed group completed 12 correct sequences per training trial with 10 s breaks between trials. The spaced group completed four correct sequences per training trial with 30 s breaks. After 108 completed sequences during training, there was a 15-minute rest wherein participants both groups performed a distraction task of double-digit addition. Before the test trials, participants completed a single warmup sequence and then had a break. Afterwards, both groups performed 15 test trials with four sequences per trial and 30 s breaks. All other aspects of the methods were identical to those of Experiment 1.

### Data analysis and cleaning

The dependent measure was time in milliseconds to complete a correct sequence. Keypress latency within sequence was measured as the time (in milliseconds) between consecutive keypresses. To reduce noise in the data, we log-transformed the keypresses latencies. The mean of the logged keypresses was then calculated for each sequence and participant. We then exponentiated those means and multiplied them by the number of correct keypresses per sequence (five in Experiment 1; nine in Experiment 2) to obtain a measure of sequence RT in milliseconds. The first completed sequence was removed from each trial prior to further analysis due to the consistently longer RTs on those sequences, indicative of warmup^1,2^.

Data were analyzed in R^71^ (version 4.2.1) using the *tidyverse*^72^ (version 2.0.0) and *BayesFactor*^73^ (version 0.9.12–4.2) packages. For Bayes factors, the Cauchy prior width was set to *r* = 0.707^74^. Interpretation followed Raftery’s guidelines^75^: 1–3 = weak, 3–20 = positive, 20–150 = strong, > 150 = very strong evidence for the null or alternative hypothesis. BF₀₁ denotes evidence for the null hypothesis; BF₁₀ denotes evidence for the alternative.

To fit Bayesian mixed-effects models, we used *brms*^76^ with sequence RT as the outcome. For numerical stability, all continuous variables were z-scored prior to modeling; reported effects are back-transformed to milliseconds for interpretability. Bayes factors comparing nested models were obtained by bridge sampling the marginal likelihoods of the scaled models, taking the median of multiple repetitions.

We conducted an unbiased data cleaning procedure to remove poor performing participants. Although our task design only allowed participants to advance after correctly executing the sequence multiple times in a trial, brief lapses in attention - such as stepping away from their keyboard - could still occur, inflating error rates and RT. Therefore, we summed each participant’s total incorrect keypresses and excluded anyone whose error count exceeded 2.5 standard deviations above the group mean. In parallel, we calculated each participant’s average RT across all trials and removed those whose mean RT fell above 2.5 standard deviations of the group mean.

## Data Availability

All data are available at [https://osf.io/ukwf9/](https:/osf.io/ukwf9) . Further information and requests for resources should be directed to and will be fulfilled by the corresponding author, TCR ( [trickard@ucsd.edu](mailto: trickard@ucsd.edu) ).

## Code availability

All stimuli, and analyses are available at https://osf.io/ukwf9/. Further information and requests for resources should be directed to and will be fulfilled by the corresponding author, TCR (*trickard@ucsd.edu*).
